# The Great War: VI. The Progress of the Sick (concluded)

**Published:** 1918-05-04

**Authors:** 


### THE GREAT WAR.

### VI.—THE PROGRESS OF THE SICK (*Concluded*).

### Motor Ambulance Convoys.

The transport of sick from field ambulances to clearing stations is carried out by motor ambulance convoys, each consisting of fifty cars. The whole of the battle front between the field ambulance and clearing station lines is mapped out into suitable areas, to each of which a convoy is allotted, so that evacuation of sick goes on continuously, undisturbed by the constant movement of troops into and out of the areas. The convoys are under the administration of the "armies," a perfect system which enables a reserve to be maintained for immediate use in any direction, and which facilitates the diversion of cars from quiet sectors to busy ones according to necessity.

### The Sick at the Clearing Stations.

Clearing stations, although mobile units, are more stationary than field ambulances, and are consequently more elaborately equipped; they also have the advantage of the services of nursing sisters. Their main rôle is to clear the Front of wounded and to treat those unfit to be moved to the Base, but when not fully employed in this way they are able to retain cases of sickness likely to recover in a fortnight or three weeks; their tenure, however, is precarious, for at the scent of battle they are evacuated to make room for the wounded.

### At the Base.

At the Base there is settled rest for a certain time for all—spring beds, feather pillows, white sheets, nursing sisters, and, above all, tranquillity. Those who are likely to be able to return to the ranks in a reasonable time are treated locally until fit for transfer to a convalescent camp; the remainder, when fit for the voyage, go back to "Blighty."

